# Use of Humanized Mouse Models for Studying HIV-1 Infection, Pathogenesis and Persistence

**Published:** 2020

**Authors:** Matthew Weichseldorfer, Alonso Heredia, Marvin Reitz, Joseph L. Bryant, Olga S. Latinovic

**Affiliations:** 1 Institute of Human Virology, School of Medicine, University of Maryland, Baltimore, United States; 2 Department of Medicine, School of Medicine, University of Maryland, Baltimore, United States; 3 Department of Pathology, School of Medicine, University of Maryland, Baltimore, United States; 4 Department of Microbiology and Immunology, School of Medicine, University of Maryland, Baltimore, United States

**Keywords:** HIV-1 infection, combined antiretroviral therapy (cART), humanized mouse models

## Abstract

Despite decades of intensive basic and clinical research efforts, there is still no successful vaccine candidate against human immunodeficiency virus (HIV-1). Standard combined antiretroviral therapy (cART) has been successfully developed and has given remarkable results suppressing HIV-1 infection and transmission. However, cART cannot fully clear the virus from the infected patients. A cure for HIV-1 is highly desirable to stop both the spread of the virus in humans and disease progression in HIV-1 patients. A safe and effective cure strategy for HIV-1 infection will require appropriate animal models that properly mimic HIV-1 infection and advance HIV-1 cure research. Animal models have been a crucial tool in the drug discovery process for investigation of HIV-1 disease mainly in preclinical evaluations of antiretroviral drugs and vaccines. An ideal animal model should recapitulate the main aspects of human-specific HIV-1 infection and pathogenesis with their associated immune responses, while permitting invasive antiretroviral studies. The best humanized mouse models would allow a thorough evaluation of antiretroviral strategies that are aimed towards reducing the establishment and size of the HIV-1 reservoirs. In this review, we evaluate multiple humanized mouse models while presenting their strengths and limitations for HIV-1 research. These humanized mouse models have been tailored in recent decades and heavily employed to address specific quintessential and remaining questions of HIV-1 persistence, pathogenesis and ultimately, eradication.

## Introduction

Different humanized mouse models have been introduced to enable HIV-1 research *in vivo*, which has been made possible by the development of immunodeficient mouse strains. Because of logistical, administrative, and ethical restrictions when working with human donor tissue samples, development of new antiretroviral therapies requires suitable small animal models that accurately mimic the natural course of HIV-1 infection in humans. In general, there is also a limited availability of human tissues (especially, Central Nervous System [CNS]) at various stages of the disease, which have been treated with different regimens, and untreated samples. Despite significant contributions of non-human primate models to HIV-1 therapeutic design and vaccine research, these animal models are not fully suitable for *in vivo* HIV-1 research due to ethical problems and high maintenance costs. Moreover, SIV infection is an inexact model for HIV-1 infection [1]. To overcome these issues, there have been extensive efforts in the last three decades to design functional, humanized immunodeficient mouse models as a tool that can properly mimic HIV-1 infection and pathogenesis *in vivo*. Therefore, immunodeficient mice engrafted with vital human cells and tissues, i.e., “humanized mice”, have become an increasingly popular and utilized option in a variety of HIV-1 preclinical studies. Different technical steps have been used to generate versatile humanized mice, including injection routes, mouse age, irradiation sources, and immunodeficient mouse strains. In this review, we describe three of the most used mouse models for HIV research.

## Hu-SCID and NSG Mouse Models

Nude mice, first developed in the mid-1960s [2], carry a mutation in the gene encoding the fork head box protein N1 (*Foxnl*^*nu*^), which results in abnormal fur growth and an athymic phenotype due to defects in thymic stromal development [3]. These mice do not produce mature CD4^+^T and CD8^+^T- cells, and therefore were not useful for HIV-1 research but were useful for xenotransplantation studies.

The first two humanized mouse chimeric hu-SCID models were designed by engrafting the SCID mice 1) with adult human PBLs [4] and 2) with human fetal thymus or liver tissue [5,6] (Table 1). The model’s susceptibility to HIV-1 infection and replication proved to be of overall benefit for studying HIV-1 infection and the efficacy of antiretroviral therapies or neutralizing antibodies [7,8], despite its inability to generate a human immune response due to lack of the full spectrum of immune cells. cART treatment of these infected hu-SCID mice resulted in renewed thymopoiesis and a normal distribution of T-cell receptor variable gene families (Table 1). HIV-1 infection distorted thymic tissue but did not stop new T-cell generation [9], with cART preventing new viral replication. This model accurately mimics acute HIV-1 infection with rapid CD4^+^T-cell loss [10], with a main benefit being that it can be infected soon after the human cells are injected [11]. The next effort in mouse humanization created the hu-PBL-NSG mouse model [12]. This involved engrafting human peripheral blood mononuclear cells (PBMCs) into adult NSG (NOD [non-obese diabetic]) SCID (Severe Combined Immunodeficiency Gamma) that have profound immune deficiency [13].

The overall efforts using humanized mice include either gene deletions or backcrosses of strains with mutations that affect T-cells, B-cells, natural killer (NK) cells, macrophages, TLRs, cytokines, and transcription factors. These mutations are meant to minimize the presence of murine cells and enhance the presence of human donor cells and tissues to better mimic human immune responses [14–16]. The original transfer of the *scid* mutation into a mouse of non-obese diabetic (NOD) background resulted in the creation of NOD-*scid* mice missing T-, B-, and NK- cells, which allowed a slightly more complete level of human cell reconstitution [17–19]. Greater success using humanized mice for HIV-1 studies were achieved by introducing a mutant interleukin 2 receptor α (*IL2rα*) gene into NOD-*scid* mice, creating NOD-*scid*-γc^null^ mice (NSG or NOG mice). These mice exhibited defective responses to mouse cytokines IL-2, IL-4, IL-7, IL-9, and IL-15 [20–22]. Knock-out of recombination activating gene (RAG) 1 or 2 (*RAG1*^*null*^ and *RAG2*^*null*^) caused more efficient immunodeficiencies including an absence of T-, B-, and NK- cells and moderately impaired dendritic cell (DC) and macrophage cell subsets [23,24]. Human cell engraftment in these mice requires neither advanced surgical methodologies nor irradiation of host animals, which makes it a simple experimental model for short-term *in vivo* studies (especially HIV entry and its inhibition). The model is also attractive for viral kinetic studies *in vivo* due to the strain-dependent differences in kinetics of HIV-1 replication in these mice. This model has been heavily utilized for short-term HIV suppression studies (3 to 4 weeks), but has serious issues, including the development of lethal xenogeneic graft-versus-host disease (GvHD) syndrome and a requirement for a more compromised mouse strain. An upgraded model showed higher human cell engraftment levels [25,26], but its remaining issue is its high variation [27,28].

The requirement for longer-term studies involving HIV-1 persistence and latency resulted in the creation of first the CD34^+^ hematopoietic stem cell (HSC) engrafted adult irradiated NSG/NOG mice, then later of the newborn RG, NSG or NOG mice. The newborn version was very attractive due to a much longer human cell lifespan, the superior lasting vitality of originally engrafted human cells [29–31], and the fact that newborn mice are already undergoing vigorous hematolymphoid expansion [32]. HIV-1 infected, CD34^+^ engrafted newborn humanized NSG mice mimic critical features of human HIV-1 infection due to generated primary immune responses and the development of a lymphoid-like system of human origin with T- and B- cells, monocytes, plasmacytoid and conventional DCs, lymph nodes, and thymic nodes. The lymphoid tissue contains key latent reservoirs established by HIV-1 during acute infection, opening new options in HIV-1 latency research, including overcoming issues when studying Gut-Associated Lymphoid Tissue (GALT) and Central Nervous System (CNS) infections. The model appears suitable to explore viral distribution in tissues, to sort cell populations to better define virus-cell interactions, and to test new antiretroviral and adjunctive therapies [33,34] in long-term studies. The new model provides an opportunity to evaluate the chronic effects of HIV-1 on the human immune system and provides human target cells and tissues in a physiologically relevant setting. Indeed, Berges *et al.*, [35] showed that RAG-hu mice sustained long-term chronic HIV-1 infection for more than a year (63 weeks).

We and others have previously shown that humanized NSG- CD34^+^ adult mice undergo productive infection and manifest several aspects of T-cell pathogenesis, indicating a good utility of this model for various new therapeutic interventions [29,30,36–38]. This model is expected to provide the fastest and most effective means with which to test various and combined antiretroviral treatments both orally and via injections. The success of antiretroviral therapies tested with this model could thus lead to important clinical applications in humans. As mentioned, this modification of the adult NSG mouse, (based upon the injection of human HSC into newborn NSG mice), supports extended end point experiments and allows successful multi-lineage development of human immune cells. Both requirements are needed for more complex *in vivo* studies including optimized antiretroviral therapies. NSG mice transplanted with CD34^+^ cells are useful for the replication of HIV-1 induced pathology and testing different antiviral therapies. This model provides a much more accessible experimental system for studies of viral infection than do models that depend upon transplanting human liver, thymic tissues, and autologous CD34^+^ HSC (BLT mice). And, as noted above and detailed in Table 1, transplanted mice develop a lymphoid-like system of human origin with T- and B- cells, monocytes, plasmacytoid, and conventional dendritic cells- DCs, mouse lymph nodes, and thymic nodes, all required for an adaptive human immune response. More than 40% of T-cells are of a naïve phenotype [39]. The ability to reconstitute a relatively representative human immune system in newborn NSG mice engrafted with HSC will surely help facilitate a better understanding of HIV-1 latency and offer the possibility of testing new treatments that could lead to an HIV-1 cure. An additional advantage of the neonatal model compared to the adult NSG model for long-term studies is a longer remaining life span (>12 months) [35,40,41].

## BLT Mouse Model

The bone-marrow-thymus tissues (derived from the same fetal donor)-subrenal capsule transplantation (BLT) model is the most robust and effective current model for HIV-1 studies because these mice have excellent T-cell reconstitution and closely recapitulate a primary immune response against productive HIV-1 infection [18,25, 42,43]. The first BLT model used NOD*scid* mice; a more recent BLT model uses upgraded mouse strains (NSG, NOG, or RG mice) [44,45]. They can be used to monitor the effects of long-lasting cART to fully diminish viremia and to prevent secondary HIV-1 transmission [46,47]. The BLT mice allow human HLA restricted T-cell responses in contrast to hu-HSC mice because of T-cell maturation in the autologous human thymus [48]. Further advantages of the BLT model are higher susceptibility to HIV-1 infection, easily controlled viremia upon cART treatment, and the advantage of using huPBMCs purified from mice and induced to express virus *ex vivo* (once viremia is reestablished after stopping cART). Importantly, unlike the CD34^+^T-NSG model, due to robust development of human mucosal immunity (including Peyer’s patches and GALT), the BLT mice are infectable by HIV-1 via vaginal, rectal, and oral routes [47,48,49]. This susceptibility to mucosal HIV-1 transmission allows exploration of different HIV-1 transmission prevention approaches such as topical HIV-1 drugs [50] (Table 1). BLT mice are heavily utilized in HIV-1 latency studies due to their ability to maintain T- lymphocytes and macrophages, as well as their ability to develop HIV-specific CD8^+^T-cell immune responses (which closely mimic human immune responses), leading to rapid viral evolution, predominantly in CD8^+^T-cell epitopes [51]. The BLT mouse model also has been used to quantify the *in vivo* replication spectrum of viruses isolated from untreated HIV+ patients with undetectable viral loads (the so-called elite controllers), showing that these viruses are replication competent and capable of infecting and depleting CD4^+^T-cells *in vivo* [52]. Unlike hu-PBL-SCID models, this model provides an environment that is more typical of the later stages of HIV-1 infection in humans (with primary cellular and humoral immune responses present and complemented with a slower CD4^+^T-cell decline).

## Limitations of the hu-Mice Models

Besides the higher costs or the mouse humanization procedures, the NSG models have some technical limitations that disadvantage their broader use in HIV-1 research. Not all animals have the engraftment levels required for successful studies. There is a remaining innate immunity that impairs engraftment, and development of GvHD (a life-terminating complication) can occur. There is an existing difficulty in generating human humoral immune responses leading to class switching and immunoglobulin G (IgG) antibody production. The adult NSG model has a functional limitation. Its lack of multi-lineage human hematopoiesis limits HIV-1 studies to a few weeks’ duration, but that issue has been partially overcome with newer hu-mouse models.

In the neonatal NSG model, some animals may develop more B- than T-cells, rendering those mice useless for HIV-1 studies. Additional issues include the possibility of lacking available mice for control experiments due to timing that includes the several months required for the engraftment, followed by the engraftment evaluation (CD45^+^ and CD4^+^T-cell numbers). Neonatal NSG mice have impaired development of the germinal centers and lymph nodes, important tissues when studying HIV-1 persistence and latency [53,54]. Despite generation of a primary immune response in CD34^+^T-NSG mice, the antibody responses are quite weak and largely restricted to immunoglobulin M (IgM) due to inefficient class switching, specifically after virus infection [28,55,56]. The model undergoes immature cell differentiation and the rare differentiation of certain cell lineages derived from HSCs [57].

The main obstacle with the BLT mouse model is the laborious surgical manipulation and time–consuming human cell reconstitution required, which leads to higher costs. A second issue is that the model cannot be used to study humoral immunity involving B-cells. Lastly, due to ethical issues related to abortion, the model cannot be used in countries such as Japan (and perhaps more recently in the United States).

## Conclusions

Further improvements of existing hu-mouse models and more accurate mimicking of the full human immune system are still needed. To improve the earlier listed limitations of the existing hu-mouse models, there are ongoing efforts towards generating new strains of immunodeficient mice transgenic for human HLA molecules. The rationale is that this will strengthen immune responses and the expression of human cytokines and growth factors and thereby improve human cell reconstitution and homeostatic maintenance. There is a need for the inclusion of other human genes responsible for cell differentiation and intracellular interactions. Expression of human growth factors and HLA proteins may help to derive a composite recipient mouse strain having all the desirable features needed for HIV research.

Numerous investigators worldwide continue searching for molecular mechanisms to resolve remaining defects in humanized mice, aiming for superior engraftment to overcome the existing deficiencies described in this review. This includes enhancement of human adaptive and innate immunity, and reduction of mouse innate immunity. Mouse models are faster and cheaper than non-human primate models and avoid concerns about reciprocal Fc-FcR evolution, i.e., how effectively human antibodies can drive Fc-mediated effector function in non-human primates. Given the notable progress made using humanized mice for HIV-1 research in recent years, the development of more advanced humanized mouse models in the HIV-1 field, especially for HIV-1 vaccine studies, is of great importance.

## Figures and Tables

**Table 1:** Summary of mouse models: Hu-PBL-SCID, SCID-hu, Hu-HSC and BLT.

Procedures		Reconstituted Cells and Tissues	Benefits
Hu-PBL-SCID	PBL injected via intraperitoneal, intrasplenic or intravenous routes	Human T-cells are present in spleen peripheral blood and peritoneal cavity	Good T-cell engraftment, Immediate use
SCID-hu	Human fetal thymus and liver fragments implanted under renal capsule	Thymocytes and naive T-cells; limited number of human cells present in peripheral organs and spleen	Significant T-cell lymphopoiesis
Hu-HSC	HSC injected via intrahepatic, intracardiac routes, or facial vein of newborn mice, upon irradiation	Near-complete human immune system; Cells are not HLA restricted (mouse thymus)	Multi-lineage hematopoiesis, Mucosa engraft, Some primary immune responses
BLT	Human fetal thymus and liver fragments implanted under renal capsule, upon irradiation; HSC injected (thy/liv tissue) intravenously	T-cells, B-cells, monocyte/macrophages and NK cells; Human cells present in the thy/liv implant, spleen, lung, liver, gut, peripheral blood and vaginal mucosa	Multi-lineage hematopoiesis, Mucosa engraft, Some primary immune responses
